# Potentially Inappropriate Medication Use among Underserved Older Latino Adults

**DOI:** 10.3390/jcm12093067

**Published:** 2023-04-23

**Authors:** Ebony King, Mohsen Bazargan, Nana Entsuah, Sayaka W. Tokumitsu, Cheryl Wisseh, Edward K. Adinkrah

**Affiliations:** 1 Department of Geriatrics, West Los Angeles VA Medical Center, Los Angeles, CA 90073, USA; 2 Department of Public Health, Charles R. Drew University of Medicine and Science (CDU), Los Angeles, CA 90059, USA; 3 Department of Family Medicine, Charles R. Drew University of Medicine and Science (CDU), Los Angeles, CA 90059, USA; sayakatokumitsu@cdrewu.edu; 4 Department of Family Medicine, University of California Los Angeles (UCLA), Los Angeles, CA 90095, USA; 5 Department of Clinical Pharmacy Practice, University of California Irvine (UCI), Irvine, CA 92617, USA

**Keywords:** potentially inappropriate medications, prescription medications, older adults, Latino, Beers Criteria, sleep difficulty

## Abstract

Background: Previous studies identified alarming increases in medication use, polypharmacy, and the use of potentially inappropriate medications (PIMs) among minority older adults with multimorbidity. However, PIM use among underserved older Latino adults is still largely unknown. The main objective of this study is to examine the prevalence of PIM use among underserved, community-dwelling older Latino adults. This study examines both the complexity of polypharmacy in this community and identifies associations between PIM and multimorbidity, polypharmacy, and access to medical care among this segment of our population. Methods: This community-based, cross-sectional study included 126 community-dwelling Latinos aged 65 years and older. The updated 2019 AGS Beers Criteria was used to identify participants using PIMs. We used multinomial logistic regression to examine the independent association of PIM with several independent variables including demographic characteristics, the number of chronic conditions, the number of prescription medications used, level of pain, and sleep difficulty. In addition, we present five cases in order to offer greater insight into PIM use among our sample. Results: One-third of participants had at least one use of PIM. Polypharmacy (≥5 medications) was observed in 55% of our sample. In addition, 46% took drugs to be used with caution (UWC). In total, 16% were taking between 9 and 24 medications, whereas 39% and 46% were taking 5 to 8 and 1 to 4 prescription medications, respectively. The multinomial logit regression analysis showed that (controlling for demographic variables) increased PIM use was associated with an increased number of prescription medications, number of chronic conditions, sleep difficulty, lack of access to primary care, financial strains, and poor self-rated health. Discussion: Both qualitative and quantitative analysis revealed recurrent themes in the missed identification of potential drug-related harm among underserved Latino older adults. Our data suggest that financial strain, lack of access to primary care, as well as an increased number of medications and co-morbidity are inter-connected. Lack of continuity of care often leads to fragmented care, putting vulnerable patients at risk of polypharmacy and drug–drug interactions as clinicians lack access to a current and complete list of medications patients are using at any given time. Therefore, improving access to health care and thereby continuity of care among older Latino adults with multimorbidity has the potential to reduce both polypharmacy and PIM use. Programs that increase access to regular care and continuity of care should be prioritized among multimorbid, undeserved, Latino older adults in an effort toward improved health equity.

## 1. Introduction

The American Geriatrics Society (AGS) Beers Criteria (AGS Beers Criteria) for Potentially Inappropriate Medication (PIM) use is a critical tool to guide clinician decision-making regarding medications to avoid or to use with caution (UWC) in older adults [1]. The use of PIMs in older adults contributes to functional and cognitive decline as well as higher healthcare utilization costs [2]. Thus, the Beers Criteria serves an important role in medication management in older adults and is necessary for informing future research and for encouraging deprescribing practices within the United States (US) [3].

Previous studies examining the burden of PIM usage suggest an increased prevalence of PIM use in urban, community-dwelling, minority older adults, as anticipated by the overall higher prevalence of cardiovascular risk factors observed in this population [4,5]. In a recent study examining PIM use among older African American adults, nearly half of the participants surveyed were taking PIMs. Of these, 56% of the PIMs were noted to potentially increase the risk of falls and fall-associated bone fractures [4]. A recently published systematic review noted that underlying the increased rates of PIM use in older adults is a higher prevalence of cardiovascular disease (CVD) risk factors in this population [6]. In the US, CVD is the leading cause of death in Latinos and is considered by the American Heart Association (AHA) to be a public health burden requiring culturally tailored interventions and policies in this minimally researched population [7].

Polypharmacy, defined as the use of five or more medications, is common in the elderly and contributes to both increased PIM use and adverse drug events (ADEs) [8]. In 2010, the National Health and Nutrition Examination Survey (NHANES) showed that more than one-quarter of US adults 65 years and older had polypharmacy, which was associated with increased use of cardiac and antidepressant medications [3]. Factors such as polypharmacy and management of multiple chronic comorbidities increase the likelihood of PIM use [9]. However, social determinants of health (SDOH) have also been associated with many health disparities in minority communities, including the risks of polypharmacy. For example, data reviewed from the Sacramento Area Latino Study on Aging (SALSA, 1996–2008) show lower odds of polypharmacy in individuals who were employed and higher odds in those with poor self-rated health and a high burden of chronic medical conditions [10]. In a survey of 632 participants pulled from the SALSA data set, 15.5% of Latino participants had polypharmacy [11].

There is a wide range of information regarding the prevalence of PIM use in the US elderly population. In a survey of US community-dwelling older adults (2006–2010 MEPS), which was comprised of 80% White, 9% Black, and 7% Hispanic participants, 43% of the sample took PIMs, with high rates of NSAIDs (11%) and benzodiazepines (9%) [12]. An international meta-analysis on the PIM use of 227,534 nursing home residents showed a PIM prevalence of 43% [13]. The major driving factor associated with increased risk of PIM use was the number of prescribed medications [13] (results were not adjusted for race in this study). In a more recent study of 935 community-dwelling, family practice patients (56% White, 41% Black, 3% other), the prevalence of polypharmacy and PIM use was 86% and 36%, respectively [8].

There are many clinical efforts to reduce harm in the elderly population based on investigations on PIM use in different ethnic populations and deprescribing strategies to improve outcomes. A review of 26 multidisciplinary interventions that utilized many tools including the Beers Criteria for medication management in nursing home-dwelling residents showed a decrease in polypharmacy, an increase in acceptance of pharmacy recommendations, and lower discharge medication costs in intervention groups [14]. However, while the Beers Criteria and other tools such as the Screening Tool of Older Persons’ Prescriptions (STOPP), have been identified as effective components of interventions to reduce PIM use, it has been noted that there are potential limitations in all tools such as the Beers Criteria for identifying PIM in the context of individual patient situations and providing guidance on what to do once a PIM has been identified, which may influence their adoption amongst primary care clinicians [15].

The study of underrepresented elderly medical populations is important, in the context of a growing and diverse elderly population, for the identification and alleviation of structurally determined health disparities. Research on health disparities is a national priority especially for the elderly as already noted by The National Institute of Aging (NIA) framework for minority health research [16]. The increasing literature on understudied minority older adults is essential to prepare for anticipated changes in the population. The US population of Latino adults aged 65 years and older was 4,638,966 in 2019 and is projected to grow to 19.9 million by 2060. In 2019, Hispanic Americans made up 9% of the older population. By 2060, the percentage is projected to be 21%. The number of Latino adults aged 85 and older is projected to increase from 509,096 in 2019 to 3.4 million in 2060 [17]. Furthermore, the foreign-born population 65 years and older, which numbered 7.3 million in 2018, representing 13.9% of the total older population in the United States, is expected to increase rapidly to 22.0 million (23.3% of the total older population) by 2060. To illustrate the importance of understanding PIM use in Latino immigrants, the US Census Bureau reported that 4 out of 10 older foreign-born people in the US from 2012 to 2016 were from Latin America [18]. This is consistent with a recent study that estimated that 40% of undocumented Latino immigrants in the USA will be 55 years or older by 2038 [19].

Few studies in the US examine the prevalence of polypharmacy and PIM use in the older Latino population [11,20]. To the best of our knowledge, no study has examined PIM among a community-based sample of older Latino adults. The main purpose of this study is to investigate PIM use among community-dwelling, older Latino adults using the revised 2019 Beers Criteria in order to identify significant patterns in the use of PIMs and UWC medications in this population. This will not only aid in the development of targeted deprescribing interventions but will also identify limitations in the Beers Criteria in support of its evolution and continued integration into clinical practice. This study aims to examine the prevalence of PIM use among underserved, community-dwelling, older Latino adults. This study examines both the complexity of polypharmacy in this community and identifies associations between PIM and multimorbidity, polypharmacy, as well as health care accessibility among this segment in our population. We present five case studies to offer additional insight into PIM use among our sample. These case studies illustrate the complexity of clinical scenarios as a tool for further synthetization and tailoring to training and clinical practice.

## 2. Methods

Study Population. This study is part of a larger effort to examine medication use among a sample of 165 Latinos aged 55 years and older. However, the current study used only those participants who were 65 years of age or older (126) from the study sample to examine the use of PIMs. Exclusion criteria included current residence in a skilled nursing facility, cognitive impairment, and enrollment in other clinical trials. This convenience sample of participants was recruited from 14 predominantly Latino senior centers and housing units located in underserved areas of the South Los Angeles Service Planning Area (SPA) 6, also referred to as “South Los Angeles”. SPA 6 serves the communities of Athens, Compton, Crenshaw, Florence, Hyde Park, Lynwood, Paramount, and Watts, which together comprise a population of over one million. South Los Angeles is one of the most underserved and under-resourced service planning areas in the County of Los Angeles. Nearly 34% of the residents in SPA 6 have household incomes below the federal poverty level. One out of four adults in SPA 6 has been diagnosed with hypertension and age-adjusted coronary heart disease. The age-adjusted death rate per 100,000 population related to diabetes in SPA 6 is 37.6 compared with 7.5 in the West Los Angeles Service Planning Area (SPA 5) [21].

The Institutional Review Board (IRB) of Charles R. Drew University of Medicine and Science approved this study. Written informed consent was collected from all participants. They were encouraged to consider the potential benefits of this project to society. Participants were told that the information they provide may contribute to improving minority senior population health outcomes. In addition, they received a remuneration fee of USD 50. Two research associates, a trained physician and a Master of Nursing, conducted the face-to-face interviews in a private room at participating sites. Overall, less than 5% of individuals who were approached refused to participate.

Measurements: In addition to standard items that measure demographic variables, our survey included validated instruments to document PIM usage, the number of prescription medications used, self-rated health status, the Short-Form McGill Pain Questionnaire-2 (SF-MPQ-2) [22,23], and healthcare access.

Demographics characteristics: We used age, gender, educational attainment, living arrangement, and financial strains as the covariates in this study. Educational attainment was operationalized as a continuous variable (number of years with school attendance). Higher scores indicated more years of education. We asked our participants whether they live alone or lived with others, which was analyzed.

Financial strain: This variable was measured using five items. Participants were asked, “In the past 12 months, how frequently were you unable to: (1) pay your rent or mortgage, (2) buy the clothes you feel your family should have, (3) buy the amount of food your family should have, (4) pay your monthly bills, and (5) make ends meet?” Items were on a 5-level response scale ranging from 1 (never) to 5 (always). A total “financial strain” score was calculated, with an average score of five items, ranging from 1 to 5. A high score was indicative of greater financial difficulty. The associations between perceived financial strain and various health measures show that this variable appears as part of a package of cognitions and emotions indicative of low morale or demoralization that have adverse effects on the subjective health of older Latino adults [24].

Access to medical care providers: This variable was measured using a single item. Participants were asked, “How difficult is it for you to visit your doctor when you need medical care?” Responses were on an “extremely difficult = 1” to “not difficult at all = 5” scale.

Self-rated health status: This variable was measured using a single question asking, “In general, would you say your health is (1) Excellent; (2) Very good; (3) Good; (4) Fair; and (5) Poor?” Even though this variable predicts mortality risk less well for Hispanic older adults than their non-Hispanic White counterparts, it remains a powerful measure that has been repeatedly used in large-scale national surveys and predicts mortality risk among all ethnic older adults [25].

Pain severity: Pain was measured using the four subscales outlined in the Short-Form McGill Pain Questionnaire-2 (SF-MPQ-2) [22,23]. For this 22-item instrument, participants were asked to describe some of the different qualities of pain and related symptoms that they had experienced in the past week using an 11-point numeric rating scale. Examination of psychometric properties of the SF-MPQ-2 among Hispanic and non-Hispanic White patients shows that this measure seems to be used equivalently across these two ethnic groups [26].

Sleep difficulty: This variable was measured using one item asking participants whether they have been told/diagnosed by their doctor as having sleep difficulty (no = 0 and yes = 1).

Chronic conditions: Participants were asked to report diagnoses known to be confirmed by a physician. We collected information on several medical conditions and other clinical diagnostic events including hypertension, diabetes, hyperlipidemia, arthritis, osteoporosis, thyroid disorder, recent fall (within 12 months), and recent hospitalization (within 12 months). Each participant’s self-reported history of recent hospitalization was used to identify subsequent medical conditions. Objective data including weight, height, and geriatric depression scores (GDS) were used to tabulate diagnosis for obesity (using BMI) and depression, respectively. Additionally, if a medical condition was not self-reported, the researchers utilized both noted drug indication and clinical judgment to assign a condition based on the assumed therapeutic purpose.

Medication use: Medication use was assessed using the drug inventory method. Participants were asked to bring all over-the-counter (OTC) and prescribed (Rx) medications that were taken during the two weeks prior to the interview. From each medication container label, the interviewer transcribed all relevant drug information including medication name, drug dosage, indication, and provider information. The medication assessment of this study used the methodology established by Sorensen and colleagues [27,28,29], which was used by our research team previously [30,31,32,33,34,35].

PIM use: The updated 2019 AGS Beers Criteria was used in this study to identify PIMs as follows: PIM was defined as medications that must be avoided, excluding medical exceptions, but were prescribed for the older adult. Medical exceptions per the Beers Criteria include the prescribing of PIM in the presence of certain rationalized criteria (examples: use of proton pump inhibitor (PPI) for less than eight weeks, use of non-cyclooxygenase-NSAIDS in low-risk groups (age less than 75 years old), topical low-dose estrogen use in women). Tables 2 and 3 of the Beers Criteria identify PIM by drug class and drug by drug–disease/syndrome interaction, respectively [1].

The AGS 2019 Updated Beers Criteria lists drug–drug interactions that should be avoided in older adults. These identified and grouped drug–drug interactions were also counted as one PIM unit, even if the individual drugs by themselves also qualified as PIM per the Beers Criteria [1]. In individual cases where an individual PIM created multiple drug–drug interactions, that medication was also counted as a PIM drug–drug interaction with each successive drug–drug interaction pairing. For each study participant, a cover sheet was developed summarizing all medications prescribed, medical conditions, PIM classified by drug class and drug–syndrome interaction, drug–drug interactions, and UWC medications.

Data Analysis: Our analysis had three parts. The first part was a descriptive analysis of all participants. This descriptive work reported frequency and percentages for categorical variables and means and standard deviation for continuous measures. Next, we calculated Pearson product-moment correlation coefficient to examine the bivariate correlation between the number of PIM, socio-demographic variables, and other relevant variables. Finally, multinomial logistic regression was used to investigate the risk factors (number of chronic conditions, sleeping difficulty, level of pain, etc.) associated with PIM, adjusting for demographic and access to care variables. For the multinomial logistic regression, the odds ratio (OR) and 95% confidence intervals (CIs) are reported. For the multivariate analysis, *p*-values of less than 0.05 were considered significant.

Case Studies: Participants in our study whose cases demonstrated unique characteristics were highlighted in selected case studies. The five cases identified share similarities in the complexity of polypharmacy, potentially inappropriate use, and with-caution medication use. Notably, these anecdotes also elicit discussion of potentially harmful medication beyond what is dictated in the Beers Criteria.

## 3. Results

Table 1 reports the characteristics of the study sample. This study included 126 Latino individuals who were between the ages of 65 and 95 years (mean = 73.7; SD ± 6.50). Approximately 41% of the participants were 75 years of age or older with 64% being self-reported women. Almost 36% of the sample reported living alone. Seventy-seven percent of the sample never completed high school. With regard to health status, only 16% of the sample reported their present health as excellent or very good, while 49% of all the participants reported fair or poor health. The number of reported chronic illnesses ranged from zero to twenty with the average being eight (mean = 7.9; SD = 3.90). Nearly one in five (22%) of participants reported at least eleven chronic medical conditions.

Table 2 reports that 16% of the participants were taking between 9 and 24 medications, whereas 39% and 46% were taking 5 to 8 and 1 to 4 prescription medications, respectively. Polypharmacy (≥five medications) was observed in 55% of participants. Our data indicate that over one-third of participants (forty-three) used at least one PIM. Of those taking at least one PIM, 84% (thirty-six) and 16.3% (seven) were taking one to two or two to three PIM, respectively. While the average PIM taken was 0.54, the range was from zero to three potentially inappropriate medications (Mean = 0.54; SD 0.88). A total of 68 PIMs were used by 43 individuals. The most common PIM drug class was the use of PPI at 33%, followed by drug–drug interactions, NSAIDs, and drugs for urinary incontinence at 17%, 10%, and 9%, respectively. Among the PIMs identified, the most commonly cited potential adverse reaction identifying the medication as PIM was falls at 34%, with a moderate correlation between PIM use and a history of recent falls (r = 0.22, *p* < 0.05). This was followed by C. Difficile/bone loss/fractures at 28% and anticholinergic effects, which include delirium, cognitive decline, and constipation, at 18%. No other significant correlations were noted between PIM use and medical histories of obesity or depression (r = 0.036 *p* = 0.79; r = 0.29 *p* = 0.24, respectively) or between a history of recent falls and the use of PIM with fall risks or the use of PIM with anticholinergic effects (r = 0.35 *p* = 0.07; r = 0.08 *p* = 0.07, respectively).

Forty-six percent of participants were prescribed drugs to be used with caution (UWC). The range of UWC medications taken was zero to three (mean = 0.67; SD = 0.83). A total of 84 UWC medications were used by 58 individuals. The most common UWC drug class was diuretic followed by inappropriate aspirin use (for primary prevention) at 43% and 38%, respectively. The most common UWC adverse medical reactions were major bleeding and syndrome of inappropriate antidiuretic hormone secretion (SIADH) at 39% and 59%, respectively.

### Bivariate Analysis

The Pearson product-moment correlation coefficient (r) was computed to assess the relationship between the number of PIM and each of the independent variables (Table 3). There was a strong, positive correlation between the PIM used and (1) the number of prescription medications (r = 0.44, *p* < 0.001), (2) the number of chronic conditions (r = 0.37, *p* < 0.001), and (3) self-reported sleep difficulty (r = 0.23, *p* < 0.001). However, the number of PIM used was negatively associated with access to medical care (r = −0.25, *p* < 0.05), indicating that decreases in access to medical care providers were correlated with increases in PIM use. Overall, the PIMs used did not appear to be associated with the other independent variables.

Multivariate Analysis: The outcome measure for the multinomial logistic regression was PIM use in three categories (no PIM, one PIM, and two or more PIMs). Since only a few subjects used more than two PIMs, we categorized participants that used more than two PIMs into “PIM ≥ 2”. The top section of Table 4 reports odds ratios (ORs) and a 95% confidence interval for the OR (95% CI) comparing PIM ≥ 2 vs. no PIM. The second part of this table reports the OR and 95% CI comparing one vs. no PIM. The reference variable for these comparisons was “no PIM”. We used two models to explain the variation in PIM use (Table 4).

Model One: This model includes eight independent variables plus the variable that measures the number of prescription medications to explain variation in PIM use. The multinomial logit estimate of the odds ratio shows (Table 4, column 1: PIM ≥ 2 relative to no PIM use) that if a participant were to increase their use of prescription medications by one medication, the relative risk for having at least two PIMs would be expected to increase by a factor of 1.68, given that the other variables in the model are held constant. Similarly, this table (column1) shows that if a participant reports (1) sleep difficulty, (2) one unit increases in positive assessment of self-rated health status, (3) no difficulty accessing primary care providers, and (4) one unit increases in financial strains, the relative risk of being in the ≥two PIM group (compared with no PIM) would be 6.95, 0.23, 0.39 and 2.19 times more likely (given that the other variables in the model are held constant), respectively. Similarly, the second part of Table 4 (Model One, column 1: one PIM relative to no PIM use) shows that given a one-unit increase in prescription medications, the relative risk of being in the one PIM group would be 1.30 times more likely, given that the other variables in the model are held constant. In addition, we observed a similar correlation between financial strains, assess to a primary care provider, and use of PIM.

Model Two: This model used the same eight independent variables; however, it excludes the number of Rx and substitutes this variable with the participants’ number of chronic conditions to avoid multi-collinearity between the independent variables. We detected a very similar association between PIM and the number of chronic conditions and Rx. The multinomial logit estimate for the odds ratio shows (Table 4, column 4: PIM ≥ 2 relative to no PIM use) that if a participant were to report one additional chronic condition, the relative risk of having at least two PIMs would be expected to increase by a factor of 1.70, given that the other variables in the model are held constant. Similarly, participants with sleep difficulty are more likely (OR = 5.49; *p* < 0.05) than their counterparts with no sleep difficulty to use at least two PIMs. Similar to Model One, financial strains, access to a primary care provider, and self-rated health all showed significant associations with PIM use.

## 4. Case Studies

Case 1: This participant is in her 70s with 16 medical conditions including DM2, depression, obesity, neuropathy, and a recent fall. She is taking a total of twelve medications, of which two are PIMs, one is a UWC, and the others are missed opportunities to identify further risks of falls. First, like many other participants, she has a long-term prescription for a PPI, which increases the risk of fracture. She is taking diphenhydramine, which though presents as a PIM for anticholinergic effects, is not rationalized in the Beers Criteria as also conferring risks of falls. With a history of recent falls, CNS active medications become PIMs due to risks of ataxia and recurrent falls. Though gabapentin is identified as a PIM in this case, she is also taking a serotonin antagonist and reuptake inhibitor (SARI) antidepressant, trazodone, and an anticholinergic, which, despite their CNS effects, are not listed as CNS active agents in the Beers Criteria. This misses the opportunity to qualify not only two additional drug-syndrome PIM interactions between recent fall and trazodone and diphenhydramine, but also misses a potential > three CNS drug–drug interaction between gabapentin, diphenhydramine, and trazodone, which further increases the risks of falls and fractures.

Case 2: This participant is in her 70s with a total of five medical conditions including liver cirrhosis and arthritis. She is taking a total of nine medications, of which two are PIMs and two are UWC. She is taking two NSAIDs, i.e., naproxen and meloxicam. While these drugs qualify as PIMs independently per drug class criteria (Beers 2019 Table 1) [1], they do not present as a drug–drug interaction. In the elderly, particularly those with potential coagulopathy such as our participant with liver cirrhosis, this might miss an opportunity to identify a significant bleeding risk. The participant is also taking two UWC medications, Hydrochlorothiazide (HCTZ) and Triamterene, which increases the risks of SIADH and hyponatremia. This highlights the severe risks of even UWC medications, particularly depending on the severity of participant co-morbidities, such as in this participant with end-stage liver disease.

Case 3: This participant is in her 60s and has a total of 12 medical conditions including obesity, urinary incontinence, and an unspecified sleep disorder. She is taking seven medications, three of which are PIMs and one is a UWC medication. She is taking two anticholinergic medications, i.e., oxybutynin and meclizine, which by themselves are PIMs due to high anticholinergic effects such as dry mouth, delirium, and constipation. Together, these two medications are a drug–drug interaction that increases the risk of cognitive decline. She is also taking one UWC medication, i.e., HCTZ, which increases the risk of SIADH/hyponatremia. Again, the deliriogenic effects of anticholinergics are not reflected within fall risks in the Beers Criteria. The polypharmacy and PIM use observed in this younger elderly patient puts her at significant risk for cognitive impairment and falls.

Case 4: This participant is in his 70s with a medical history notable for nine medical conditions including depression, hypertension, and heart disease. He is taking seven medications, of which only one, i.e., low-dose aspirin, qualifies as potentially harmful, being a UWC medication. He is also prescribed oxycodone and fentanyl. Without a recent history of a fall, opiates by themselves do not qualify as PIMs. Oxycodone and fentanyl also do not qualify as drug–drug interactions, despite presenting as drug–drug interactions when taken with benzodiazepines and gabapentin with the adverse reaction of severe sedation, respiratory depression, overdose, and death. The participant has no clear medical indication for taking opioids. The participants’ use of opiates has strong potential harm, although this is not reflected in the Beers analysis.

Case 5: This participant is a female in her late 60s with a medical history significant for hypertension, hyperlipidemia, and back pain. She has a total of 13 self-reported medical conditions and 9 drugs, 2 of which are PIMs, including 1 drug–drug interaction. The participant was taking losartan 100 mg dated from September 2019 to September 2020 with three refills and is also taking a combination of amlodipine/benazepril 10/4 mg dated from October 2019 to October 2020 with four refills. The simultaneous use of both angiotensin-converting enzyme inhibitors (ACEis) and angiotensin II receptor blockers (ARBs) poses a therapeutic risk of hyperkalemia. Per the Beers Criteria, there is moderate evidence and a strong recommendation to avoid this drug combination in patients with severe chronic kidney disease, which was assumed in this participant’s medication profile.

## 5. Discussion

Polypharmacy and drug-related harm are considerable challenges in the management of multimorbid older adults [36,37]. Polypharmacy contributes to an increased risk of mobility issues, frailty, falls, adverse drug reactions, long-term placement, and mortality, making deprescribing policies an important target for the care of older adults [37]. Polypharmacy is a major driver for potentially inappropriate medication use [35]. NHANES data from 1988 to 2010 looking at the prevalence of potentially inappropriate medication use in the US population shows mostly White cohorts are prescribed PIMs at a rate of 15% [3]. In contrast, studies with selected minority cohorts show a considerable disparity in increased PIM rates, where in a more recent study of elderly Black Americans using the 2015 AGS Beers Criteria, nearly one in two (46%) Blacks were prescribed PIMs [4]. NHANES data from 2011 to 2018 shows that Blacks and Latinos experience cardiovascular risk factors at rates higher than non-Hispanic Whites [38]. In our study, consequently, we hypothesized that Latinos would have high rates of polypharmacy and PIM use, similar to those observed in the Black population.

Our data show that more than half of our study cohort (55%) experienced polypharmacy. Of these, 29% with polypharmacy took more than eight medications (range: nine to twenty-four), 94% had at least one CVD risk factor, and 34% took at least one PIM. This rate is lower than more recent data with an estimated national average of PIM use over 50% [39] and lower than the rates observed in the Black subjects in our previous study, where 70% experienced polypharmacy and 46% experienced PIM use [4]. Studies demonstrate that despite the higher prevalence rate of CVD risk factors, the Latino population paradoxically experiences lower rates of CVD prevalence and mortality compared to non-Hispanic Whites [7]. Given that this is one of the first studies to examine PIM use in the Latino population, further studies are required to determine if similar PIM use and related adverse outcomes are observed among Latinos.

The multinomial logit regression analysis shows that when controlling for demographic variables, increased PIM use was associated with a lack of access to primary care providers, financial strains, an increased number of prescribed medications, and an increased number of chronic conditions. Financial strains, a lack of access to primary care, as well as an increased number of medications and co-morbidity, are inter-connected. It is well-established that patients who live in medically underserved areas have a higher incidence of preventable emergency department (ED) utilization [40]. In addition, the lack of continuity of care is associated with a greater risk of frequent ED utilization [41]. Therefore, it is reasonable to assume that the lack of continuity of care may force underserved older Latino adults to more frequently utilize the ED or urgent care facilities, leading to fragmented care and documentation gaps, putting vulnerable patients at risk of prescribing cascades, polypharmacy, and drug–drug interactions. This is because providers may not have a current list of prescribed medications that patients are using at any given time nor a full picture of the circumstances that led to said drugs being prescribed nor their intended duration of use (i.e., acute vs. chronic use). Older adults presenting to the ED often have multiple complex medical conditions and take numerous medications, yet deprescribing in the ED can be challenging because it requires careful medication reconciliation to guide medication adjustments as well as ongoing support and follow-up [42]. Therefore, improving continuity of care among older Latino adults has the potential to reduce the disparities in polypharmacy and PIM use. Continuity of care and equitable access are ongoing and complex challenges faced by our healthcare system. Improving access in medically underserved areas requires a multi-pronged approach which includes increased efforts to recruit clinicians to practice in medically underserved areas from identification and recruitment of students who originate from underserved communities to increased training programs integrated into medically underserved areas. Efforts to improve access should also include investment in programs that improve care coordination and patient navigation. This is particularly important in immigrant populations where language concordance is often lacking.

Our data also show that, when controlling for demographic variables, increased PIM use was associated with sleep difficulty among our sample of underserved Latino older adults. Both our qualitative (case studies) and quantitative analyses echo previous research suggesting an urgent need for a medication review to identify and reduce PIMs among older adults [43]. Appropriate management of chronic insomnia is crucial and prescribing hypnotic/sedative drugs is common among older adults. Given that the Beers Criteria suggests avoiding all benzodiazepines in older adults to treat insomnia, clinicians often resort to use of sedatives/hypnotic medications. Stricter adherence to evidence-based guidelines is essential for more discretionary use of hypnotics and sedatives given their potential harm among older adults [44]. Indeed, the absence of behavioral resources to address sleep difficulties may drive PIM use of sedative and hypnotics in minority underserved older adults [44]. Improving medication reconciliation and identifying medications that may contribute to disrupted sleep is essential, as well as access to behavioral interventions that address sleep disturbance such as cognitive behavioral therapy for insomnia (CBT-I) have the potential to impact sedative/hypnotic use in underserved older adults. The role of the clinical pharmacist in interdisciplinary teams is especially highlighted in this regard. Clinical pharmacists with specialized training in caring for the older adult, given their medication expertise, can be utilized in the healthcare team to further investigate the safety, efficacy, and appropriateness of each medication prescribed to the older adult. Clinical pharmacists conducting medication reconciliations can identify potentially harmful drug–drug interactions, drug–disease state interactions, and other pharmacokinetic concerns which are especially relevant to the care of the older adult. We recommend continuous advocacy for policy changes that would recognize the unique contributions of the pharmacist to the healthcare team. Specifically, approval of prescriber status and fair reimbursement rates are recommended as modalities that would support the work of the pharmacist in this capacity.

This study is novel in being one of the first to characterize UWC medication use in the elderly minority and Latino populations. Despite the lessened warning severity, in our study, the use of UWC medications was observed to potentially result in serious adverse reactions, including the risk of SIADH and major bleeding events at 60% and 39%, respectively. Hyponatremia is the most common electrolyte disorder detected in the elderly and has been shown to be significantly associated with falls [45], reinforcing the need to prioritize the risk category warning of these drugs. Given the absence of hospital records, we were unable to calculate the incidence of major bleeding events in our cohort. At 38%, aspirin for primary prevention was the second most common UWC drug class in our cohort. Many meta-analyses of CVD primary prevention trials show a 50–60% increased incidence of major gastrointestinal or extracranial bleeding in older adults who take aspirin for primary prevention, a risk level that only increases with advancing age [46]. Future studies are needed to determine if these adverse medical events are similarly distributed in this population.

The qualitative analysis of our case studies revealed repeated missed opportunities to quantify even more potential for drug-related harm. For example, in case 1, a woman in her early 60s taking both aspirin for primary prevention and chronic ibuprofen did not get flagged as taking a PIM or UWC due to the participant’s younger age (per the Beers Criteria medical exceptions of <75 years old and less than 70 years old, respectively).

Furthermore, concurrent use of multiple NSAIDs is not recognized as a PIM drug–drug interaction in the Beers Criteria [1]. This is particularly concerning in case 2, in which a similar pattern of duplicate NSAID use was observed in a patient with a history of liver cirrhosis. Consequently, in this and in similar cases, major bleeding risks are not accurately identified, rendering patients at a greater risk of experiencing this alarming adverse drug event.

Medication-related falls are a major contributor to morbidity and mortality in the elderly [47]. In our study, 33% of PIMs taken increased the risks of falls and fall-associated fractures. Similarly, the qualitative analysis of our case studies points to the missed opportunity to identify relevant medication interactions that increased the risk of falls and fall-associated fractures in our cohort. For example, in one case in our study, a participant with a history of vertigo was prescribed an opiate, which due to the participant’s syncope and history of recent falls, could arguably be captured as a drug–syndrome interaction that increases the risk of subsequent falls. Additionally, anticholinergics and muscle relaxants, despite their potential central nervous system (CNS) altering and subsequent fall risks, are not identified as such in the Beers Criteria [48]. The 2019 AGS Beers CNS active medications include antidepressants such as TCAs, SSRIs, SNRIs, antipsychotics, antiepileptics, nonbenzodiazepine/benzodiazepine receptor agonists, hypnotics, and opioids [1]. Not reflecting anticholinergics and muscle relaxants in this fall-risk category means these medications, when taken together or with other Beers classified CNS active agents, are subsequently unable to be qualified as a >3 CNS active drug–drug interaction, which per Beers Criteria, increases the risks of falls and fractures [1]. The use of PIMs and in particular anticholinergics has been shown in prior studies to increase the rates of fracture-specific hospital admissions [48]. Reflecting more saliently the fall risks associated with these and other CNS-affecting drugs within the Beers Criteria would aid in their identification by medical providers and reduction of potential harm.

As mentioned previously, the Beers Criteria and other tools such as the Screening Tool of Older Persons’ Prescriptions (STOPP), have been critiqued for not considering the clinical context in which PIMs are prescribed for older patients and not providing guidance for clinicians once PIMs are identified. To improve utilization of this invaluable tool, it is recommended that future editions of the Beers Criteria include alternative medication recommendations for PIMs to assist clinicians in the medical management of common issues such as sleep disturbance to minimize risk to older adults.

## 6. Strengths and Weaknesses

The strengths of this study include a multidisciplinary team of investigators, including a gerontologist with expertise in health service research among minority older adults, as well as physicians and clinical pharmacists with expertise in geriatric medicine. This is notable as older adults often have several comorbid conditions and require an interdisciplinary care model to optimize their care. This multidisciplinary model was exemplified in this study. One of the primary limitations of our study was the research team’s inaccessibility to participants’ medical records. As survey responses were self-reported by participants, the research team was unable to retrospectively validate the medications patients reported taking or the indication for the medications used. It is important to note that the data collected regarding medication-related information in the parent study were collected directly from the medication bottles that participants provided. Some medications were classified as PIMs due to prolonged use beyond what is recommended. For example, PPI use at >8 weeks, “chronic” use of NSAIDS, and metoclopramide at 12 weeks. The intended duration of medications was not clearly indicated in several cases. As such, the research team estimated long-term and chronic medication use by reviewing medication count and refill information. In addition, this is an observational survey subject to selection bias.

Some PIMs in the AGS 2019 Beers Criteria are classified by interaction as medications requiring dose adjustments per the recipient’s glomerular filtration rate (GFR) [1]. In this study, the researchers did not have access to laboratory records and therefore were unable to capture these PIMs, with the exception of unique cases where medications were indicated for end-stage renal disease (ESRD).

Additionally, data were collected in 2018, and the Beers criteria rendition used in this study, released in 2019, was the latest at the time of this writing. This poses a unique challenge as the 2019 rendition contained several changes compared to its predecessor, which was released in 2015. These changes include the removal of medications that were no longer available in the US and medication-related problems that were not considered sufficiently unique to the older adult population. Of note, the 2019 Beers Criteria states that these omissions are not an implication that these high-risk medications are safe for use in older adults [1]. This amendment to the Beers Criteria, therefore, limits its use as a solitary comprehensive tool for the identification of potentially harmful medications in older adults and could result in clinically relevant adverse events for the older adult. Furthermore, given that data were collected in 2018, they do not reflect potential adjustments in prescribing practices providers would make based on the future version of the Beers Criteria.

## 7. Conclusions

We believe the results of this study provide relevant and vital information to inform an understudied area within this vulnerable population. This study also demonstrates the utility of the Beers Criteria to evaluate complex medical profiles. The evaluation of polypharmacy and detection of PIM use remains an important and necessary skill in geriatric assessment and deprescribing policies [14,49]. The Beers Criteria have traditionally been released in three-year increments; however, due to the COVID-19 pandemic and other factors, the next rendition of the Beers Criteria, due in 2021, has been delayed. Even with anticipated updates, it is important to remember that the Beers Criteria might leave gaps in the detection of adverse medical events and is just one clinical tool, which is not necessarily comprehensive, to guide medication deprescribing. This is especially true when taking care of underserved populations in which increased medical complexity and structural disparities impact access to regular care and adherence to medical recommendations [11,50]. Decisions regarding medication utilization in the elderly should be based on a holistic methodology including shared decisions [4] that integrate tools such as the Beers Criteria with considerations for functional status, social context, and overall personal goals of care. Frameworks such as the American Geriatrics Society 5M’s can be a particularly helpful tool in conceptualizing important factors that contribute to the health and wellness of older adults.

A recent systematic review of the literature published between 2008 and 2018 on deprescribing practices using the STOPP criteria show that nearly all deprescribing interventions were delivered by pharmacists [14]. These interventions targeted an array of outcomes but were generally positive in impact, associating pharmacy-led deprescribing interventions with decreased hospitalization, cumulative exposure to medications, and medication-related falls [14]. A more recent review of studies published in 2020 shows that interventions with multi-disciplinary teams including geriatricians, dieticians, nurses, and occupational therapists are also successful in improving indices of polypharmacy in the elderly [51]. However, the homogeneity in the population demographics in these studies requires more targeted research on the impact of deprescribing interventions in the older Latino and African American populations, which tend to have greater medical morbidity, polypharmacy, and structural barriers that attenuate optimal medical care [4,10,11].

Racial health disparities extend and even worsen throughout a lifetime into advanced age. With our aging minority population, we can anticipate the need for more targeted research and interventions to address the health outcomes in these vulnerable populations. Addressing health disparities requires not only dedicated research in underserved communities but also the integration of validated tools and guidelines with constructs of social determinants and health equity that impact care. In contribution to this goal, the analysis of our previously studied elderly Black cohort [4] and now our elderly Latino cohort provides a landscape to understand the challenges related to prescribing practices in these communities with implications for future research and intervention.

## Figures and Tables

**Table 1:** Demographic characteristics and health status of the sample (N = 126).

	*n*	%
Gender		
Male	46	36.5
Female	80	63.5
Age		
65–74	75	59.5
≥75	51	40.5
Education		
No high school diploma	96	77.0
High school diploma	29	23.0
Live Alone		
No	81	64.3
Yes	45	35.7
Sleep Difficulty		
No	85	72.6
Yes	32	27.4
Self-rated Health		
Excellent/very good	19	16.2
good	41	34.7
Fair/poor	58	49.1
		Mean ± SD
Age (years: 65–95)		73.7 ± 6.50
Financial Strains (1–5)		3.5 ± 1.46
Self-rated health (1– 5)		3.29 ± 0.98
Number of chronic conditions (0–20)		7.9 ± 3.90
Number of RX (0–17)		5.08 ± 3.80
Level of pain (average of 22 items: 0–10)		2.03 ± 2.36

**Table 2:** Medication Use, Polypharmacy, PIM, and Drug–Drug Interaction.

Medication Use	N (%)	Mean
Number of Prescribed Medications		
0–4	58 (46.0)	
5–8	38 (38.1)	5.1 (0–17)
9–24	20 (15.9)	
Polypharmacy ≥ 5	69 (54.7)	
Potentially Inappropriate Medication Use		
Yes	43 (34.1)	
No	83 (65.9)	
PIM 1–2	36 (83.7)	0.56 (0–3)
PIM 3–4	7 (16.3)	
Major Drug-Drug Interactions		
Yes	12 (9.5)	
No	114 (90.5)	
Drugs to be Used with Caution		
Yes	63 (50)	
No	63 (50)	
Inappropriate Aspirin Use (for primary prevention)		
Yes	32 (25.4)	
No	94 (74.6)	

**Table 3:** Correlation matrix (Person’s r) between dependent and independent variables.

	1	2	3	4	5	6	7	8	9	10
PIM	1.00									
2. Age	0.017	1.00								
3. Gender	0.147	−0.124	1.00							
4. Education	−0.059	−0.169	0.045	1.00						
5. Financial Strains	0.005	0.113	−0.221 *	0.122	1.00					
6. Access to Care	−0.252 *	−0.054	−0.243 **	−0.067	0.392 **	1.00				
7. Self-Rated Health	−0.118	−0.148	0.020	−0.113	−0.298 **	−0.089	1.00			
8. Sleep Difficulty	0.228 *	−0.114	0.059	−0.031	−0.275 **	−0.151	0.308 **	1.00		
9. Level of Pain	0.072	−0.057	0.120	0.068	−0.538 **	−0.220 *	0.311 **	0.425 **	1.00	
10. N of RX	0.439 **	−0.019	0.060	−0.038	−0.103	−0.063	0.153	0.075	0.191 *	1
11. N of Chronic Conditions	0.373 **	−0.094	0.184 *	0.044	−0.347 **	−0.175	0.432 **	0.450 **	0.406 **	0.568 **

** Correlation is significant at the 0.01 level (2-tailed). * Correlation is significant at the 0.05 level (2-tailed).

**Table 4:** Multinomial logistic regression: parameters estimates. Odds ratio (OR), 95% confidence intervals of the OR, and *p*-values.

PIM (≥Two vs. No PIM) ^a^	Model 1 ^b^			Model 2 ^c^		
Independent Variable	OR	95% CI	Sig.	OR	95% CI	Sig.
Age	0.92	0.81–1.04	0.166	0.93	0.83–1.05	0.242
Gender	3.65	0.68–19.7	0.133	2.02	0.41–9.94	0.388
Education	0.64	0.36–1.14	0.133	0.61	0.36–1.04	0.071
Financial Strains	2.19	1.01–4.87	0.050	1.67	0.91–3.07	0.098
Access to Medical Care	0.39	0.17–0.90	0.027	0.52	0.26–1.07	0.076
Self-Rated Health	0.23	0.09–0.55	0.001	0.21	0.08–0.51	0.001
Sleep Difficulty	6.95	2.24–18.6	0.006	5.49	1.11–19.0	0.049
Level of Pain	1.12	0.74–1.69	0.607	0.88	0.60–1.27	0.489
Number of RX	1.68	1.31–2.17	0.001	N/A	N/A	N/A
N of Chronic Conditions	N/A	N/A	N/A	1.70	1.27–2.27	0.001
**PIM (One vs. No PIM)** ^a^						
Age	1.03	0.94–1.13	0.537	1.03	0.94–1.13	0.509
Gender	1.15	0.34–3.89	0.828	1.13	0.34–3.74	0.844
Education	0.74	0.44–1.26	0.269	0.68	0.40–1.15	0.149
Financial Strains	2.43	1.18–5.03	0.016	2.68	1.33–5.37	0.006
Access to Medical Care	0.34	0.15–0.72	0.005	0.35	0.17–0.73	0.005
Self-Rated Health	1.25	0.61–2.55	0.544	1.22	0.59–2.54	0.593
Sleep Difficulty	1.59	0.38–6.66	0.529	0.98	0.25–3.82	0.979
Level of Pain	1.03	0.72–1.49	0.864	1.01	0.70–1.46	0.959
N of RX	1.30	1.08–1.57	0.006	N/A	N/A	N/A
N of Chronic Conditions	N/A	N/A	N/A	1.24	0.99–1.56	0.060
-2 Log Likelihood	138.06 (DF: 18; *p* < 0.0001)			148.83 (DF: 18; *p* < 0.0001)		
Pseudo R-Square Nagelkerke	0.517			0.450		

Note: ^a^ The reference category is “No Potentially Inappropriate Medication” (PMI). ^b^ Model 1 includes the number of Rx and excludes the number of chronic conditions from the independent variables list to avoid multi-collinearity. ^c^ Model 2 includes the number of chronic conditions and excludes the number of Rx from the list of independent variables.

## Data Availability

The data sets used and analyzed in the current study are available from the corresponding author for collaborative studies. Personal identification details of the participants were separated from the completed questionnaires. The data were stored in a locked room at the Charles R. Drew University of Medicine and Science (CDU). No information relating to identifiable individuals was disseminated at all. The data sets used and analyzed in the current study are available from the corresponding author for collaborative studies.
